# Statistics of Suicide in France

**Published:** 1846-03

**Authors:** 


*Statistics of Suicide in France.*—The number of suicides in 1843 amounted to 3020; which surpassed that of 1842 by 154; of 1841, by 206; of 1840, by 263; so that there has been a regular increase during the last three years. The distribution of suicides in the different departments is as follows:

| | | | |
|---|---|---|---|
| Seine, - - | 551 | Somme, - - | 71 |
| ——et Oise, - | 113 | L'Herault, - - | 13 |
| ——Inferieure, | 112 | Haute Garonne, | 12 |
| Marne, - - | 101 | Le Gard, - - | 23 |
| Nord, - - | 80 | La Gironde - | 26 |
| L'Aisne, - - | 78 | L'Isre, - - - | 30 |
| Seine et Marne, | 75 | Rhone, - - | 44 |

Of these there were of females, 729, or 24 in the 100. We remark also, 15 of children under 16 years of age; 20 octogenarians; 170 septuagenarians; and 384 sexagenarians. The months most prolific in self-destruction were May, June and July. The most common mode of death was drowning: 1098 adopted this; 954 hanged or strangled themselves; 450 used fire-arms; and 206 destroyed themselves with the fumes of charcoal. Of these latter, 151 were of the department of the Seine, in which Paris is situated. The motives were mostly disappointed love, jealousy, the consequences of debauchery, reverse of fortune, domestic sorrow, and physical suffering. One quarter of them were not of sound mind at the time.—*Revue Medicale.—Ibid.*

